# Odor Pleasantness Modulates Functional Connectivity in the Olfactory Hedonic Processing Network

**DOI:** 10.3390/brainsci12101408

**Published:** 2022-10-19

**Authors:** Veit Frederik Kepler, Manuel S. Seet, Junji Hamano, Mariana Saba, Nitish V. Thakor, Stavros I. Dimitriadis, Andrei Dragomir

**Affiliations:** 1 Department of Biomedical Engineering, National University of Singapore, 4 Engineering Drive 3, Singapore 117583, Singapore; 2 The N.1 Institute of Health, National University of Singapore, 28 Medical Drive, Singapore 117456, Singapore; 3 International Operations, Procter & Gamble, 70 Biopolis Street, Singapore 138547, Singapore; 4 Department of Biomedical Engineering, Johns Hopkins University, Baltimore, MD 21205, USA; 5 Department of Clinical Psychology and Psychobiology, Faculty of Psychology, University of Barcelona, Passeig de la Vall d’Hebron, 171, 08035 Barcelona, Spain; 6 Institut de Neurociències, University of Barcelona, Campus Mundet, Edifici de Ponent, Passeig de la Vall d’Hebron, 171, 08035 Barcelona, Spain; 7 Integrative Neuroimaging Lab, 55133 Thessaloniki, Greece; 8 Neuroinformatics Group, Cardiff University Brain Research Imaging Centre (CUBRIC), School of Psychology, College of Biomedical and Life Sciences, Cardiff CF24 4HQ, UK; 9 Division of Psychological Medicine and Clinical Neurosciences, School of Medicine, College of Biomedical and Life Sciences, Cardiff University, Cardiff CF24 4HQ, UK; 10 Department of Biomedical Engineering, University of Houston, 3517 Cullen Blvd, Houston, TX 77204, USA

**Keywords:** electroencephalography, brain connectivity, olfaction, hedonic evaluation, lateralization

## Abstract

Olfactory hedonic evaluation is the primary dimension of olfactory perception and thus central to our sense of smell. It involves complex interactions between brain regions associated with sensory, affective and reward processing. Despite a recent increase in interest, several aspects of olfactory hedonic evaluation remain ambiguous: uncertainty surrounds the communication between, and interaction among, brain areas during hedonic evaluation of olfactory stimuli with different levels of pleasantness, as well as the corresponding supporting oscillatory mechanisms. In our study we investigated changes in functional interactions among brain areas in response to odor stimuli using electroencephalography (EEG). To this goal, functional connectivity networks were estimated based on phase synchronization between EEG signals using the weighted phase lag index (wPLI). Graph theoretic metrics were subsequently used to quantify the resulting changes in functional connectivity of relevant brain regions involved in olfactory hedonic evaluation. Our results indicate that odor stimuli of different hedonic values evoke significantly different interaction patterns among brain regions within the olfactory cortex, as well as in the anterior cingulate and orbitofrontal cortices. Furthermore, significant hemispheric laterality effects have been observed in the prefrontal and anterior cingulate cortices, specifically in the beta ((13–30) Hz) and gamma ((30–40) Hz) frequency bands.

## 1. Introduction

Hedonic evaluation is integral to our sense of smell, and, in this context, subjective pleasantness is a primary dimension in the perceptual characterization of olfactory stimuli [1]. Even though humans are remarkably capable at discriminating between odors differing by small molecular variations [2]—owing to the sheer diversity of receptor types inhabiting the olfactory epithelium [3]—the ability to verbally identify odors is relatively poor. Yet, humans can readily and reliably appraise odors in terms of their pleasantness, so much so that pleasantness/hedonic tone is the most prominent descriptor organizing the semantic space for olfactory objects [4]. Olfactory hedonic evaluation may be partially innate [5], as it would be evolutionarily beneficial to determine if a foodstuff is edible/nutritious, or if a prospective mate is compatible, based on the pleasing qualities of olfactory cues [6].

Evidence obtained via structural and functional neuroimaging also demonstrates the centrality of hedonic processing in olfactory perception. There is considerable overlap in the functional neuroanatomy underlying olfactory, affective and reward processing [7]. After chemosensory registration of the odorant at the epithelium, olfactory neural signals are conveyed to the olfactory bulb and then to the primary olfactory cortex (OLF), which mainly encodes the identity [8] and sensory attributes (e.g., intensity/concentration) of the olfactory stimulus [9]. The olfactory cortex then projects to two major centers of affective/reward processing—the amygdala and the orbitofrontal cortex (OFC). The amygdala rapidly and automatically processes the emotional content of sensory stimuli [10]. The OFC is the key hub that codes for odor pleasantness [11], as well as reward [12], and has direct bidirectional structural connections with the amygdala [13]. The OFC projects to the medial prefrontal cortex (mPFC), which supports explicit evaluation of stimulus-based emotional [14] and reward value [15] underlying value-guided decision-making. The OFC also projects to the anterior cingulate cortex (ACC), which processes the rewarding value of stimuli and facilitates reward-driven learning and action [16].

Despite these advances, there exist several issues that demand further inquiry. First, while it is understood that olfactory hedonic processing is implemented across multiple brain areas, it is unclear how these brain areas communicate and interact with each other during online hedonic evaluation of odors. Furthermore, olfactory hedonic processing occurs on a fast timescale, with cortical responses occurring as soon as 250 ms following stimuli, and further cognitive and behavioral responses unfolding within several hundred milliseconds [17]. Neuroimaging research on the topic, based mostly on functional magnetic resonance imaging (fMRI), has been limited in exploring fast temporal scale activity. Moreover, such studies often rely on analyzing neural activation patterns of individual brain areas [18,19], with a dearth of publications directly examining functional connectivity among them. The majority of previous efforts utilizing EEG [20,21,22,23] also lack this focus. However, deeper insights into olfactory hedonic processing can be drawn via network-based connectivity analyses that complement prior activation studies. In this direction, the handful of olfaction studies employing EEG functional connectivity analysis are focused on limited characterizations of global changes to the connectivity network in response to pleasant and unpleasant stimuli, or without an investigation of the relevant brain regions using graph theoretic metrics [24,25]. Other efforts focused on collateral cognitive processes (not hedonic processing), such as attention to stimuli [26] or pathological aspects [27]. In previous works, we investigated cortical integration mechanisms by assessing cross-frequency hubs [28] and functional network communities [29]. However, these studies were limited to investigating specific frequency bands.

Second, uncertainty still surrounds the lateralization of hedonic processing in the brain. Hedonic appraisal of positive experiences has been thought to implicate the left hemisphere more so than its right counterpart [18,30]. However, some studies have found a right-hemispheric bias in neural activation [31,32], while others report no discernible lateralized effects [33]. Therefore, it is of interest to revisit the issue of laterality in pleasantness evaluation—with respect to olfactory stimulation—this time approaching from the perspective of connectivity.

Third, particular relevance pertains to the application of functional connectivity analysis in the context of stimuli featuring the same valence level (the hedonic tone of an event). Previous studies [20,21,34] compare pleasant to unpleasant stimuli. However, since valence tends to dominate olfactory perception [35], it is preferable to compare pleasant-only stimuli with different levels of pleasantness. This prevents extraneous influences of binary valence (i.e., pleasant/unpleasant) on neural activity. These three points mark the main contributions of the present work.

The objective of this study is to analyze functional connectivity of the olfactory hedonic network during exposure to exclusively pleasant olfactory stimuli. Electroencephalography (EEG) was chosen due to its high temporal resolution. This allows unraveling of the fast timescales at which olfactory hedonic responses occur, as well as scrutinizing oscillatory mechanisms involved at different frequency bands. EEG signals were recorded while participants were presented with exclusively pleasant odors (fragrances); these were rated on pleasantness and intensity immediately after each presentation. The EEG data underwent source localization to uncover the activity of five selected brain areas—the amygdala, OFC, mPFC, ACC and the olfactory cortex—whose involvement in olfactory processing has been discussed previously. Functional connectivity and graph theoretic analyses were subsequently performed in an exploratory manner in the delta ((1–4) Hz), theta ((4–8) Hz), alpha ((8–13) Hz), beta ((13–30) Hz) and gamma ((30–40) Hz) frequency bands, as prior literature documents significant EEG modulations within these ranges during olfactory perception [36,37]. To our knowledge, these efforts present the first EEG-based study investigating functional connectivity during the hedonic evaluation of exclusively pleasant olfactory stimuli.

It was hypothesized that fragrances of different levels of pleasantness (high vs. low) elicit significantly different network connectivity patterns across brain areas, which are quantified in terms of standard metrics (provided by graph theory), and across brain hemispheres. Elevated connectivity may be found in both left- and right-hemisphere brain areas, respectively, as previous literature on the topic presents non-uniform conclusions.

## 2. Materials and Methods

This section outlines the experimental design, collection and processing of data, as well as the construction of pertinent functional connectivity networks. Subsequently, the mathematical and statistical frameworks underlying relevant analyses are introduced.

### 2.1. Participants

Twenty-one female subjects aged 21 to 45 were recruited. Exclusion criteria consisted of respiratory dysfunctions, neurological disorders, disorders affecting the olfactory system, or presence of any metallic implants that could affect data quality. Approval of the experimental protocol was granted by the Institution Review Board (IRB) of the National University of Singapore (study reference code N-18-051). Subjects received monetary remuneration for participating in the study.

### 2.2. Experimental Design

Subjects were blindfolded to avoid the influence of any visual stimuli on brain activity and equipped with a nasal respiration sensor for the monitoring of breathing. The experiment was conducted in a temperature-controlled, isolated laboratory room to prevent noise and other confounding influences from affecting data acquisition and the presentation of odors. Four olfactory stimuli consisting of exclusively pleasant fragrances (as prepared by a perfumery professional) were used, with subjective pleasantness and intensity being monitored via behavioral questionnaires. Subjects were presented one fragrance at a time over a trial period lasting 8 s. The experiment comprised a randomized sequence of ten trials for each fragrance, totaling 40 exposures. Following each trial, subjects were asked to instantly rate the pleasantness and intensity of the presented stimulus on a scale of 0 to 10. The inter-trial interval was kept at 2 min and—to avoid odor masking—coffee beans were presented to the subjects after each trial, following completion of stimulus rating [23]. The behavioral questionnaire scores were then considered to identify the two fragrances with the highest and lowest pleasantness ratings, respectively. Only the 20 corresponding trials (10 each for the high- and low-pleasantness stimulus) were used for further data analysis.

### 2.3. EEG Data Acquisition & Preprocessing

EEG signals were recorded using a standard 64-channel ANT-Neuro cap at a sampling rate of 512 Hz using Ag/AgCl electrodes. Conductive electrolyte gel was applied between the scalp and the electrodes; impedance was kept below 5 kΩ. The EEGLAB toolbox [38] was used to preprocess the raw data, first down-sampling it to 256 Hz and then applying a bandpass filter (zero-phase Butterworth type filter, 4th order) from 1 Hz to 40 Hz. Signal artifacts caused by muscle movements were removed via automatic artifact rejection (AAR) [39]. EEG signals were subsequently re-referenced to the common average. Corresponding to the trial length (8 s), the data was then epoched to extract segments of 1 s each, and independent component analysis (ICA) was conducted to remove eye movement related artifacts [40]. For this purpose, the FastICA algorithm was run in a per-subject manner by concatenating all trials of a subject. The ICLabel classifier was then used to inform artifactual independent components rejection, with a threshold level of 90% confidence [41].

### 2.4. Functional Connectivity Network Construction

**Figure 1:**
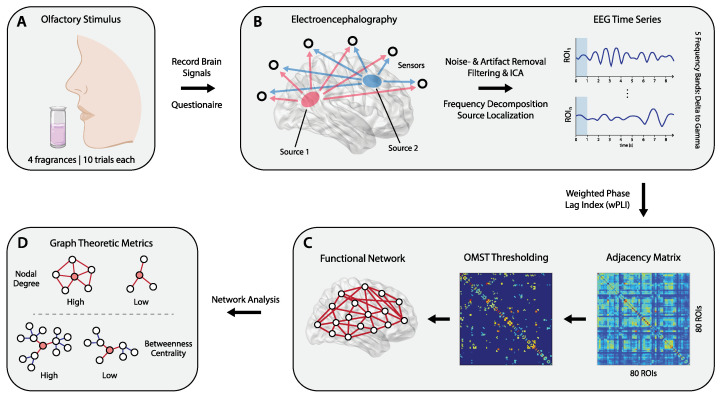
Overview of the study’s methodology. EEG signals recorded in response to pleasant olfactory stimuli exposure (**A**) were preprocessed and source localization was performed to estimate the cortical sources generating scalp level signals (**B**). Then, functional connectivity between source-level EEG signals was estimated using the weighted phase lag index (wPLI), yielding 80×80 adjacency matrices that store pairwise connectivity values between sources (ROIs). The wPLI matrices were subject to further thresholding based on orthogonal minimum spanning trees (OMST) (**C**), resulting in functional connectivity networks that were analyzed (**D**) to estimate graph theoretic metrics (nodal degree, clustering coefficient, betweenness centrality). Using said metrics, statistical analyses were carried out to quantify cortical dynamics induced by olfactory stimuli of different perceived pleasantness. This included direct comparisons of experimental factors via paired samples *t*-tests, as well as utilizing graph metrics to assess hemispheric laterality effects.

Subsequently, source localization was performed using standardized-Low Resolution Electromagnetic Tomography (s-LORETA) to estimate the cortical sources generating the recorded scalp level EEG signals [42]. The s-LORETA software extracted voxel-based activation information, which was further parceled into established anatomical regions of interest (116 ROIs), based on the Automatic Anatomical Labeling (AAL) atlas [43]. ROIs corresponding to cerebellar and sub-cortical regions were discarded, resulting in a set of size n = 80. To construct functional connectivity networks from the EEG time series, each ROI was considered as a node, with individual functional connections between them as edges. These connections were calculated using the weighted phase lag index (wPLI), which estimates functional connectivity based on the phase-synchronization of current source density values [44], and has the advantage of being less sensitive to noise and volume conduction effects [45]. As seen in Figure 1, the result is a 80×80 adjacency matrix. Figure S1, located in the Supplementary Materials, shows a more detailed representation of network construction from EEG time series. Functional connectivity networks were constructed for the following frequency band-ranges: delta ([1–4] Hz), theta ([4–8] Hz), alpha ([8–13] Hz), beta ([13–30] Hz) and gamma ([30–40] Hz).

Afterwards, Dimitriadis’ orthogonal minimum spanning tree (OMST) algorithm [46] was used for topological filtering of said matrices; this thresholding procedure has the advantage of yielding reproducible networks [47]. At the same time, compared to traditional sparsity thresholding, applying OMSTs yields more biologically relevant functional networks by not differentiating weak from strong connections. They also retain the advantage of minimal spanning trees, as resulting networks contain no isolated nodes [48].

### 2.5. Graph Theoretic Metrics & Analysis

To characterize neural responses to pleasant fragrance stimuli, several graph theoretic metrics were estimated, including the weighted nodal degree (WD), betweenness centrality (BC) and clustering coefficient (CC). These can capture changes in the configuration of functional connectivity networks in response to different stimuli, both in terms of localized, as well as distributed processing [49].

As elaborated by Fornito et al. [49], the nodal degree is a commonly used metric to characterize localized, segregated functional activity, by means of quantifying the number of each ROI’s functional connections. The weighted degree, by extension, also incorporates the strength of these connections, by summing the weight of the edges a node is connected to [49]. Sporns [50] describes how the betweenness centrality characterizes integrated (distributed) brain functional activity by means of estimating how well a given ROI is embedded within its functional network. BC estimation is based on the proportion of shortest paths along which an ROI is located, compared to the total number of shortest paths in the network. Lastly, the clustering coefficient, defined as the probability of finding a connection between any two neighbors of a given node [50], describes the “cliquishness” of a typical neighborhood and measures network integration/segregation [51].

The aforementioned graph metrics were computed for both pleasantness conditions (averaged across the respective 10 high and low pleasantness trials) for each subject. Paired samples *t*-tests were subsequently used to identify statistically significant differences between high- and low-pleasantness stimuli within individual regions. Results were corrected for multiple testing in accordance with the procedure introduced by Storey [52]; the *a priori* probability was set at 0.05. To avoid any extraneous influence of multiple sniffs on cortical activity—see Kareken et al. [53]—all statistical analyses were confined to the first 1000 milliseconds of fragrance exposure and as such solely within the first sniff. The one-second window was chosen because it is long enough for odor-evoked cortical responses to manifest [54], but still within the duration of an average sniff (≈1.6 s [55]).

### 2.6. Laterality Analysis

In addition to direct comparisons of graph metrics across subjects, the functional prevalence of one hemisphere over the other was investigated between pleasantness conditions. To do so, graph metrics were used to compute a *laterality coefficient*, defined as:

(1) $$C_{L}=\frac{{\mathrm{metric}}_{\mathrm{left}}-{\mathrm{metric}}_{\mathrm{right}}}{{\mathrm{metric}}_{\mathrm{left}}+{\mathrm{metric}}_{\mathrm{right}}}$$

where “metric” denotes a given graph theoretic metric (WD, CC, BC). By definition, $C_{L}$ has range [−1 1]. When $C_{L}>1$ the result is labeled “left dominant” (LD) and, vice versa, “right dominant” (RD) when $C_{L}<1$. To compare laterality coefficients across pleasantness conditions (averaged over 10 respective trials for each subject), paired samples *t*-tests were used; corrections (*a priori* probability = 0.05) were performed using Storey’s method [52].

### 2.7. Olfactory Hedonic Network

**Figure 2:**
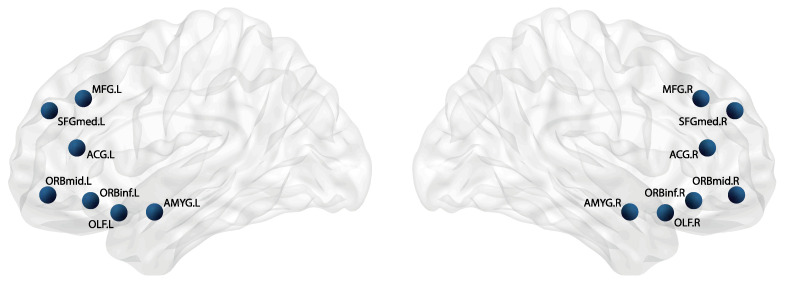
Location of selected AAL ROIs underlying major regions involved in olfactory, affective and reward processing. Regions comprise a total of 14 nodes: The middle frontal gyrus (${\mathrm{MFG}}_{L/R}$), the superior frontal gyrus, medial part (${\mathrm{SFGmed}}_{L/R}$), the anterior cingulate gyrus (${\mathrm{ACG}}_{L/R}$), the middle frontal gyrus, orbital part (${\mathrm{ORBmid}}_{L/R}$), the inferior frontal gyrus, orbital part (${\mathrm{ORBinf}}_{L/R}$), the olfactory cortex (${\mathrm{OLF}}_{L/R}$) and the amygdala (${\mathrm{AMYG}}_{L/R}$).

To investigate differences in the neural response to olfactory stimuli of different pleasantness values, and the impact on the underlying olfactory, affective and reward processing regions, further functional network analysis focused on 14 cortical source ROIs of the AAL atlas—their location in the brain highlighted by Figure 2—considered to comprise the OFC, mPFC, OLF, ACC and amygdala. These regions, as discussed in the introduction, are part of the core network that drives olfactory hedonic processing [7,15]. With respect to the lateral orientation of their constituent ROIs, they were then defined as: ${\mathrm{OFC}}_{\mathrm{Left}/\mathrm{Right}}$ = ORBmid_L/R_ + ORBinf_L/R_; ${\mathrm{mPFC}}_{\mathrm{Left}/\mathrm{Right}}$ = MFG_L/R_ + SFGmed_L/R_; ${\mathrm{OLF}}_{\mathrm{Left}/\mathrm{Right}}$ = OLF_L/R_; ${\mathrm{ACC}}_{\mathrm{Left}/\mathrm{Right}}$ = ACG_L/R_; ${\mathrm{Amygdala}}_{\mathrm{Left}/\mathrm{Right}}$ = AMYG_L/R_. Where regions were considered to consist of more than one ROI (i.e., OFC, mPFC), the average of relevant metrics was considered during analysis.

### 2.8. Null Network Comparisons

To additionally benchmark whether significant results were statistically unexpected and due to the topology of a given network, graph null-hypothesis testing was carried out. As outlined by Váša and Mišić [56], the statistical significance of a network feature *x* was evaluated by computing that same network feature on a population of “null models”, created by systematically disrupting and/or preserving characteristics of the original network. Comparing, using statistical testing, the original and “null features” then reveals whether feature *x* can be attributed to characteristics of the original network that were unaffected during the creation of null models [56].

To account for inter-subject variability, null-hypothesis testing was carried out at the trial-level. Wherever statistically significant differences in graph theoretic metrics or laterality coefficients were observed (between high and low pleasantness conditions), the *randmio_und_connected* function, taken from Rubinov and Sporns’ Brain Connectivity Toolbox [57], was used to create null models. Networks were randomized while degree distribution was preserved, with the function’s rewiring parameter set to *ITER = 10* (the approximate number of times an edge was rewired). For each of the 10 trials per subject, the network at hand was randomized a total of 500 times, with the “null-value” of the corresponding feature (graph metric or laterality coefficient) calculated each iteration; this was done for both pleasantness levels. The resulting population of 500 null-values was then averaged across the 10 trials, and – using one-sample *t*-tests—compared to the empirically obtained test-values (separately for pleasantness values and corrected using Storey’s method [52]).

Subsequently, only data that showed statistically significant differences on the trial-level was considered for further analysis. Null-values were compared to their empirical counterparts at the subject-level: the relevant feature (graph metric or laterality coefficient), computed as the average over the 500 null networks for both pleasantness levels, was further averaged across the number of trials. Finally, null-values of the features were compared to their empirical counterparts by means of paired-samples *t*-tests (false discovery rate (FDR) correction was carried out using Storey’s method [52]).

Consider the following example: the weighted nodal degree of the OLF in the beta band was found to differ significantly between pleasantness levels. After considering individual subjects at the trial-level using one-sample *t*-tests (n = 500), only those that showed significant differences were considered for subject-level comparisons: the null-value of the weighted degree (OLF) for each pleasantness condition was found by computing said metric on 500 null-networks, averaging results across the network population and with respect to the number of trials. Paired-samples *t*-tests were then used to compare between the null-feature and empirical value, comparing *within* pleasantness levels (i.e., ${\mathrm{pleasantness}}_{\mathrm{high},\mathrm{original}}$ vs. ${\mathrm{pleasantness}}_{\mathrm{high},\mathrm{null}}$).

## 3. Results

Significant outcomes regarding the hedonic evaluation of olfactory stimuli are reported in this section. This includes an interpretation of the behavioural questionnaires used to assess pleasantness, as well as results of the functional connectivity analysis using graph metrics. In addition, pertinent hemispheric laterality effects are summarized, and an assessment of relevant cortical connections is provided.

### 3.1. Behavioral Data

**Figure 3:**
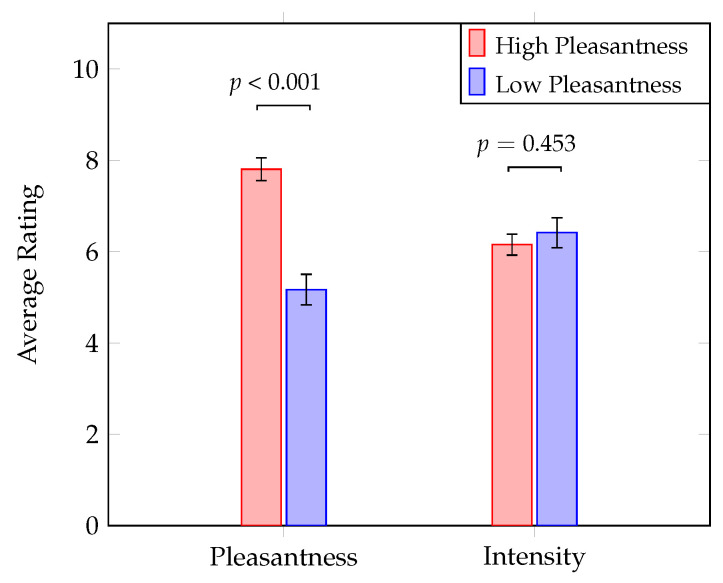
Paired-samples *t*-tests on behavioral response data reveal significant differences between high and low pleasantness stimuli; no meaningful discrepancies in fragrance intensity were observed. FDR corrected *p*-values are depicted; error bars show the standard error.

Analysis of the self-reported data revealed substantial variations in pleasantness ratings. All 21 subjects showed statistically significant differences (p<0.001) between their ratings of the highest and lowest pleasantness stimuli, respectively. Corresponding intensity scores did not differ significantly (*p* = 0.453), confirming that stimulus-related arousal (perceived intensity) was not modulated meaningfully across different levels of pleasantness. Figure 3 displays average pleasantness and intensity ratings for all subjects.

### 3.2. Graph Metrics & Functional Connectivity

**Figure 4:**
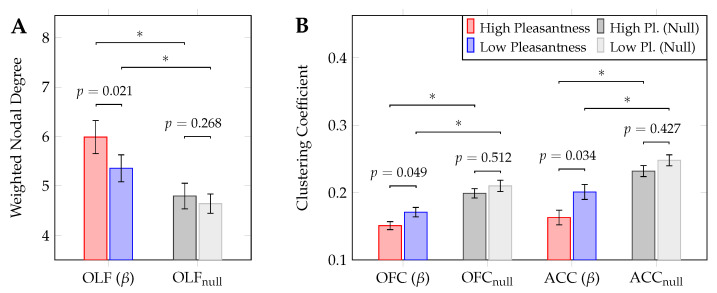
Statistically significant differences in graph theoretic metrics between high- and low pleasantness stimuli (individual regions, paired samples *t*-tests), including results of graph null-hypothesis testing (*: *p* < 0.01). Plots show results relating to the weighted nodal degree (**A**) and clustering coefficient (**B**). All events were observed in the beta (β) frequency band. FDR-corrected *p*-values are depicted; error bars show the standard error.

Across the five frequency bands, no statistically significant differences (*p* > 0.05) in graph metrics were observed when comparing the values of the metrics in the high vs. low pleasantness conditions, averaged across all 80 nodes. Considering the regions in the olfactory hedonic network (Section 2.7), three instances of such significant differences were observed. Specifically, as shown in Figure 4, the magnitude of the OLF’s weighted degree in the beta band (β) is significantly larger during exposure to high-pleasantness stimuli (p=0.021, after FDR correction). Conversely, the OFC and ACC were shown to exhibit significantly larger clustering coefficients (also in the beta band) during exposure to the low-pleasantness stimuli (p=0.049 and p=0.034, respectively; FDR corrected). 

These results underline the important role played by the OLF, OFC and ACC in mediating olfactory hedonic processing. The higher nodal degree values shown by the OLF during exposure to high-pleasantness stimuli indicate the fact that the olfactory cortex strengthens its role as a processing hub in response to these stimuli. Furthermore, the significant changes in the clustering coefficient suggest that the OFC and ACC mediate downstream olfactory processing differently across pleasantness levels. Specifically, the increased specialized (or segregated) processing observed in the case of low pleasantness stimuli (when compared to high pleasantness stimuli), highlights the crucial roles played by the OFC and ACC in olfaction.

Importantly, graph null-hypothesis testing revealed significant differences (p<0.01; *t*-test, FDR-corrected) across the entirety of comparisons between metrics computed on null-networks and their counterparts in the high- and low-pleasantness conditions.

The dichotomy informing our results (high vs. low pleasantness) stems from behavioural data (see Figure 3). To test whether stimuli pleasantness can be reliably predicted by graph theoretic metrics, binomial logistic regression was carried out for the significant instances shown in Figure 4. In all three cases (Supplementay Materials, Figure S2, panels A to C) regression analysis showed a significant relationship between outcome (pleasantness) and predictor variable (graph metrics). The weighted degree for the OLF (beta band, panel A of Figure 4) exhibited a negative relationship (odds ratio = 0.421, *p* = 0.024), while the clustering coefficient for the OFC and ACC (beta band, panel B in Figure 4) displayed positive associations (odds ratio > 1 in both cases, *p* = 0.045 and *p* = 0.030, respectively).

**Figure 5:**
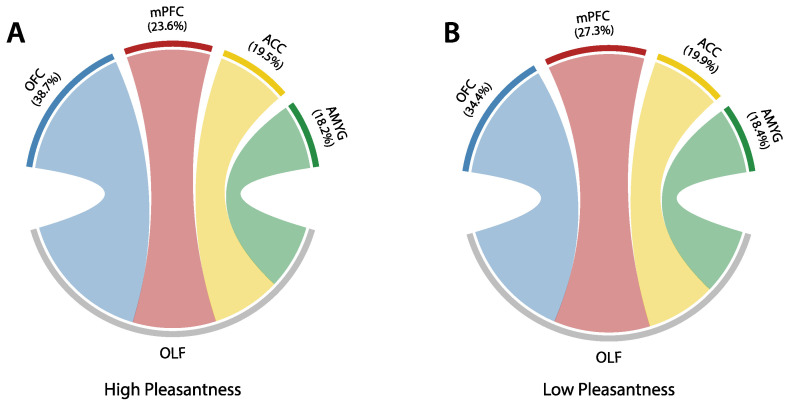
Chord diagrams outlining normalized connectivity preferences of the olfactory cortex (OLF) to regions included in the proposed hedonic evaluation model (consisting of OFC, mPFC, OLF, ACC, AMYG), for high- (panel **A**) and low pleasantness (panel **B**) stimuli. Only the beta band ([13–30] Hz) was considered.

To further evaluate hub-related functions of the OLF during olfactory perception, a qualitative analysis of its connectivity preferences within the network of selected ROIs was carried out. The weighted adjacency matrix representing said network was isolated from the original 80×80 OMST matrix and averaged across the number of trials, as well as subjects; this was done for high- and low-pleasantness stimuli. As shown in Figure 5, chord diagrams were then used to display the normalized connectivity weights between the OLF and remaining network regions. Differences across the high- and low-pleasantness stimuli for connections between the OLF and OFC (38.7% for high-pleasantness vs. 34.4% for low-pleasantness), as well as the OLF and mPFC (23.6% for high-pleasantness vs. 27.3% for low-pleasantness) are more pronounced than those between the OLF and the ACC or amygdala (almost no discernible changes).

**Figure 6:**
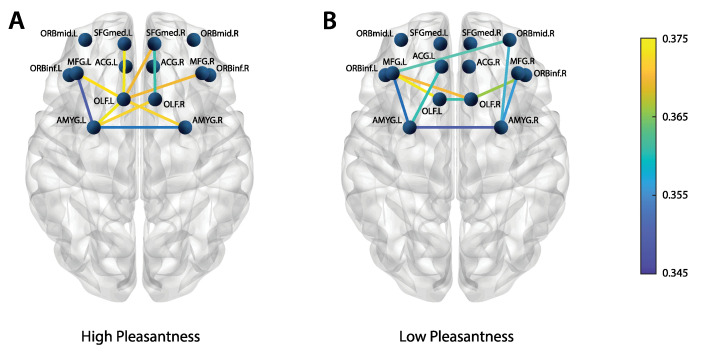
Top 10 strongest connections (from the wPLI adjacency matrices) within the hedonic olfactory model, averaged across subjects and trials. (Panel **A**) shows the high-pleasantness condition, (panel **B**) the low-pleasantness counterpart. Beta band ([13–30] Hz) results are displayed.

Apart from the OLF, the connectivity preferences for the entirety of the proposed olfactory hedonic model were assessed as well. Network edges (wPLI values) in the beta band were averaged across trials and subjects and then sorted by weight; the ten strongest connections (for both pleasantness levels) are displayed in Figure 6.

The weighted degree of the OLF is larger for the high-pleasantness stimuli (panel **A**) when compared to its low-pleasantness counterpart (panel **B**); this mirrors panel A of Figure 4. In general, the high-pleasantness stimuli appear to elicit stronger connections (i.e., larger connectivity weights). On the other hand, the low-pleasantness stimuli seem to feature more distributed connectivity patterns.

### 3.3. Laterality

**Figure 7:**
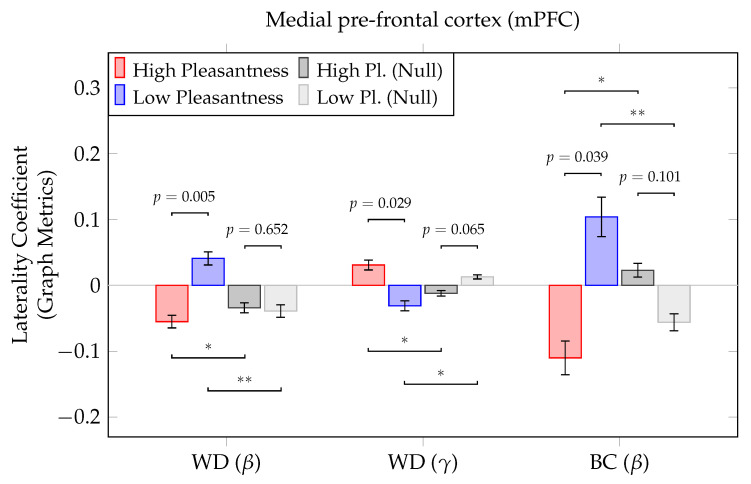
Statistically significant differences in laterality coefficients between high- and low pleasantness stimuli (individual regions, paired samples t-tests), including results of graph null-hypothesis testing (*: *p* < 0.05, **: *p* < 0.01). Coefficients were computed based on graph metrics; positive coefficients indicate a left-dominant response, right-dominance is indicated by negative coefficients. FDR corrected *p*-values are depicted; error bars show the standard error.

As in the case of graph metrics analysis, no significant differences were observed when assessing laterality effects across the entire 80 nodes (p>0.05); this was true for all five frequency bands. However, when investigating laterality effects of ROIs in the olfactory hedonic network, significant differences between the two conditions were observed. Figure 7 shows the medial pre-frontal cortex exhibiting significant differences in laterality coefficients. Specifically, in the [13–30] Hz range (beta), the low-pleasantness stimulus appeared to evoke left-dominant activity when computed using the weighted nodal degree (p=0.003; *t*-test, FDR-corrected) and betweenness centrality (p=0.039; *t*-test, FDR-corrected). Conversely, in the gamma band, the high-pleasantness stimulus elicited a left-dominant response when assessing laterality using the weighted degree (p=0.029; *t*-test, FDR-corrected).

The significant lateralization of the pre-frontal cortex, including its medial regions, has long been associated with changes in emotional and social behavior, suggesting the mPFC to play a crucial role in activating different components of emotions [58]. Our results suggest that the mPFC may also mediate olfactory and emotion processing pathways through different oscillatory mechanisms in the beta and gamma bands.

Similar to Section 3.2, significant results displayed in Figure 7 were further examined using binomial logistic regression. Once more, significant relationships between between outcome (pleasantness) and predictor variable (laterality coefficients) were observed in all three instances. Panels D to F of Figure S2 in the Supplementary Materials show these results. Laterality coefficients computed using the weighted degree and the betweenness centrality in the beta band showed a positive relationship (odds ratio > 1 in both cases, *p* = 0.006 and *p* = 0.017, respectively). When considering coefficients obtained via the weighted degree in the gamma band, a negative association was obtained (odds ratio < 1, *p* = 0.026).

## 4. Discussion

In the present study, we sought to investigate how functional connectivity among brain regions related to olfactory hedonic processing changes in response to stimuli of different levels of pleasantness. Research characterizing the neural underpinnings of olfactory processing has seen increasing interest recently, due to the significant behavioral and clinical relevance of this sensory modality. For example, the hedonic evaluation of odors has been investigated in relation to obesity [59], pain perception [60] and depression [61]. Despite a surge in research pertaining olfactory processing, important aspects related to the widely distributed nature of brain networks involved in the hedonic evaluation of odors remain to be elucidated.

Previous studies focusing on said evaluation discriminated between pleasant and unpleasant odours; a widely cited example is a study by Rolls et al. [34], which utilized fMRI. EEG has also been employed to this end; a 2014 paper by Kroupi et al. [20] and a 2020 publication by Hou et al. [21] directly used EEG signals to compare between pleasant and unpleasant odors. Another 2016 study [22] used EEG to calculate the approach/withdrawal index with regards to olfactory stimuli.

The scope of previous research lacks the distinct combination of (a) comparing pleasantness levels *within* the positive segment of the hedonic spectrum (i.e., high vs. low pleasantness as opposed to pleasant vs. unpleasant) and (b) approaching the topic from the standpoint of functional connectivity (e.g., employing the wPLI) and quantifiable graph theoretic metrics. Previous work by our lab [23,62] aimed at using EEG to compare within pleasantness levels (a), yet lacks the functional connectivity approach (b). To address this gap and add to the body of previously published work, we performed experiments in which participants were exposed repeatedly to exclusively pleasant olfactory stimuli, with neural responses being recorded using EEG. Subsequently, source localization of the EEG data was performed to estimate source-level signals and the corresponding cortical regions. Functional connectivity analysis (employing the wPLI) was then performed to model and quantify changes induced in the functional network. This was accomplished using graph theoretic metrics, which were computed across all five frequency bands (delta to gamma).

First, we found that, at the global network level (comprising all 80 cortical ROIs), there were no statistically significant differences between graph metrics when comparing between the two conditions. This reinforces our initial hypothesis that brain processing supporting olfactory hedonic evaluation is facilitated by a more localized network consisting of regions involved in sensory, affective and reward processing, as reported by other studies [19]. Second, we aimed at characterizing the impact of olfactory stimuli on the functional connectivity within this olfactory hedonic processing network. Importantly—as it indicates hub-related activities—the weighted nodal degree for the olfactory cortex was found to differ significantly during exposure to stimuli of different pleasantness values (high pleasantness stimuli elicited higher degree magnitudes). This reinforces findings by Rolls [7], as well as other studies [63,64], who mention this region to be involved in evaluating the pleasantness of odors. It must be noted that previous fMRI research suggests the dominant function of the primary OLF to be coding for the identity and sensory attributes of odors [65], further pinpointing the piriform cortex to be responsible for this task.

The multifaceted olfactory percept, of which hedonic evaluation is a major part, is shaped by projections from the OLF across predominantly higher levels of the olfactory processing hierarchy. This includes the OFC and ACC, which evaluate the pleasantness and reward value of odors [7] and may facilitate subsequent behavioral action. In this context, our findings of significant differences in localized processing at the level of the OFC and ACC, as indexed by the clustering coefficient metric, support the major role attributed to the duo in olfactory hedonic evaluation. Interestingly, we observed significantly lower levels of localized processing activity in these regions in the high pleasantness condition. We speculate that this may be due to a relative suppression of complex localized cortical activity, instead favoring interconnections of the OFC and ACC to other regions. However, this needs to be further investigated in future studies.

Importantly, significant differences in graph theoretic metrics were all observed in the beta frequency range ([13–30] Hz). Such oscillations have been previously identified to play a major role in supporting integration of various cortical areas, and across cognitive systems. The beta band has also been reported to be particularly involved in sensory perception, as well as being linked to brain mechanisms relevant to olfactory [66] and reward processing [67]. Our results underline the important role played by beta oscillations in coordinating information transfer spanning multiple neurocognitive mechanisms during olfactory hedonic perception.

Finally, a focal interest of the paper revolves around unearthing laterality effects with regards to regions encompassing the olfactory hedonic network. Functional lateralization, i.e., the differential activation of the brain’s hemispheres for achieving specialized functions in certain tasks, has been previously associated with neural processing in olfaction [68,69], as well as emotion and hedonic processing [70]. There are currently different interpretations regarding frontal lateralization. One predominant view is that asymmetric frontal activations are associated with the hedonic valence of stimuli (positive vs. negative), where left hemisphere dominance is observed in response to positive stimuli. Another view suggests that the motivational system is engaged by the stimulus (approach vs. avoidance), in which left hemisphere dominance facilitates the approach to engaging stimuli. A more recent hypothesis, which aims to account for contradictory subsequent findings, proposes a more nuanced view, in that lateralization should be viewed from a more localised perspective. Specifically, subregions of the frontal cortex are proposed to drive asymmetric activations, with support from distinct but interrelated subnetworks exhibiting their own lateralization patterns [70].

Our findings seem to support the latter hypothesis. We found high-pleasantness fragrances to elicit both left- and right-dominant lateralization across different frequency bands, especially with regards to the medial pre-frontal cortex (mPFC). In particular, the beta band ([13–30] Hz) contained a right-dominant response, with the weighted nodal degree and betweenness centrality of the right mPFC exhibiting larger magnitudes than their left-hemisphere counterparts. Conversely, the gamma band ([30–40] Hz) included a left-dominant activation of the mPFC when considering the weighted degree. These findings may suggest that different sub-networks operating at distinct oscillation modes drive the observed hemispheric lateralization. Our results also highlight the important hub-related activity of the mPFC—a key region in processing the hedonic value [71], as well as appetitive (or rewarding) value of stimuli [15].

### Limitations

Crucially, the current study was not set out to map the direction of interactions between brain areas subserving olfactory hedonic processing. Characterizing directed functional connectivity within the olfactory hedonic network (e.g., via partial directed coherence) presents the most proximate avenue for prospective future work. It is likely that many of the functional interactions considered in the study involve both feed-forward and feedback signaling acting along the reciprocal structural pathways connecting the proposed model.

Furthermore, participants recruited for this study were exclusively female; this was done for two reasons: first, to avoid heterogeneity due to gender differences, and, second, due to the previously reported difference in the olfactory ability of females and males [72]. As established by Royet et al. [73], females show increased left-based lateralization during the emotional processing of odors, especially with regards to the orbitofrontal cortex. This may present a confounding effect when investigating hemispheric laterality, especially since the OFC projects to the medial pre-frontal cortex [14,15].

## 5. Conclusions

The present study investigated how functional connectivity among brain areas involved in olfactory hedonic processing changes in response to odor stimuli featuring different levels of pleasantness. Graph metrics were used to compare key brain regions involved in the olfactory hedonic processing network and informed an investigation into laterality effects. Several significant differences regarding these regions were found, most notably including the olfactory cortex, as well as the orbitofrontal and medial pre-frontal cortices. Our results strengthen the current scientific consensus regarding the importance of the OFC and mPFC in olfactory hedonic processing, while also supporting a more recently emerging view that favors an increasingly localized understanding of hemispheric laterality.

The design of our study features three key characteristics. First, by utilizing fragrance stimuli of exclusively pleasant nature, different levels of positive hedonic evaluation were compared, in order to avoid cross-valence confounding effects; this would be the case when comparing positive vs. negative stimuli. Second, functional connectivity was assessed at the cortical source level, yielding a network of relevant brain regions which allows for accurate modeling of lateralization. Third, EEG signals were used to unearth neural activity pertaining to olfactory hedonic processing. Due to its non-invasive nature, coupled with a high temporal resolution, ease of implementation and relatively low cost, EEG presents a feasible option for brain-computer interface (BCI) applications. This is especially relevant for the field of consumer neuroscience, which relies on neural signals to discern customer preferences and make more informed decisions regarding product development. Other relevant areas include clinical applications where tools for objective affective evaluation of olfactory stimuli are needed (e.g., when investigating depression).

## Data Availability

Processed EEG data will be made available upon reasonable request.

## Supplementary Materials

The following supporting information can be downloaded at: https://www.mdpi.com/article/10.3390/brainsci12101408/s1, Figure S1: Network Construction; Figure S2: Binomial Regression Plots.

## Abbreviations

The following abbreviations are used in this manuscript:

ROI	Region of interest, a single entry in the AAL atlas
AAL	Automated anatomical labelling
OMST	Orthogonal minimum spanning tree
fMRI	Functional magnetic resonance imaging
WD	Weighted nodal degree
CC	Clustering coefficient
BC	Betweenness centrality
LD	Left dominant
RD	Right dominant
δ	Delta frequency band [1–4] Hz
θ	Theta frequency band [4–8] Hz
α	Alpha frequency band [8–13] Hz
β	Beta frequency band [13–30] Hz
γ	Gamma frequency band [30–40] Hz
