# Supplementary material for: Odor Pleasantness Modulates Functional Connectivity in the Olfactory Hedonic Processing Network
Source: Brain Sci. 2022 Oct 19;12(10):1408. doi: 10.3390/brainsci12101408 (PMC9599424; doi:10.3390/brainsci12101408)
Supplement: Supplementary file 1 [file brainsci-12-01408-s001.zip › brainsci-1941650-supplementary.pdf]

Supplementary Material

Network Construction

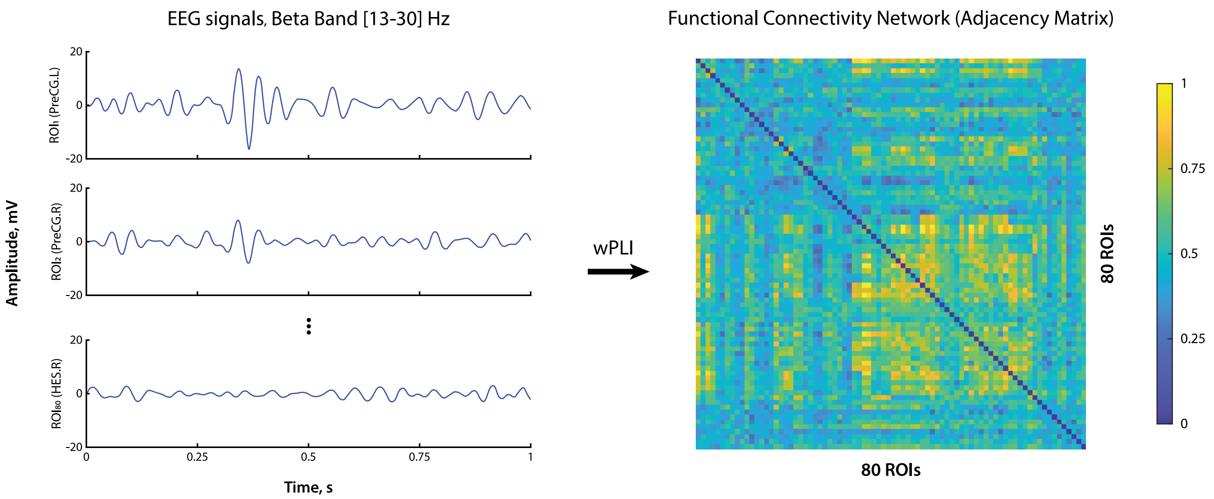

Figure S1. Simplified representation of network construction from pre-processed EEG signals; the beta frequency band ([13-30] Hz) is used as an example. Phase synchronization between signals from source localized ROIs (80 in total, based on the automatic anatomical labeling atlas) are assessed using the weighted phase lag index (wPLI). The result is a functional connectivity network represented by 80×80 adjacency matrices.

Binomial Logistic Regression

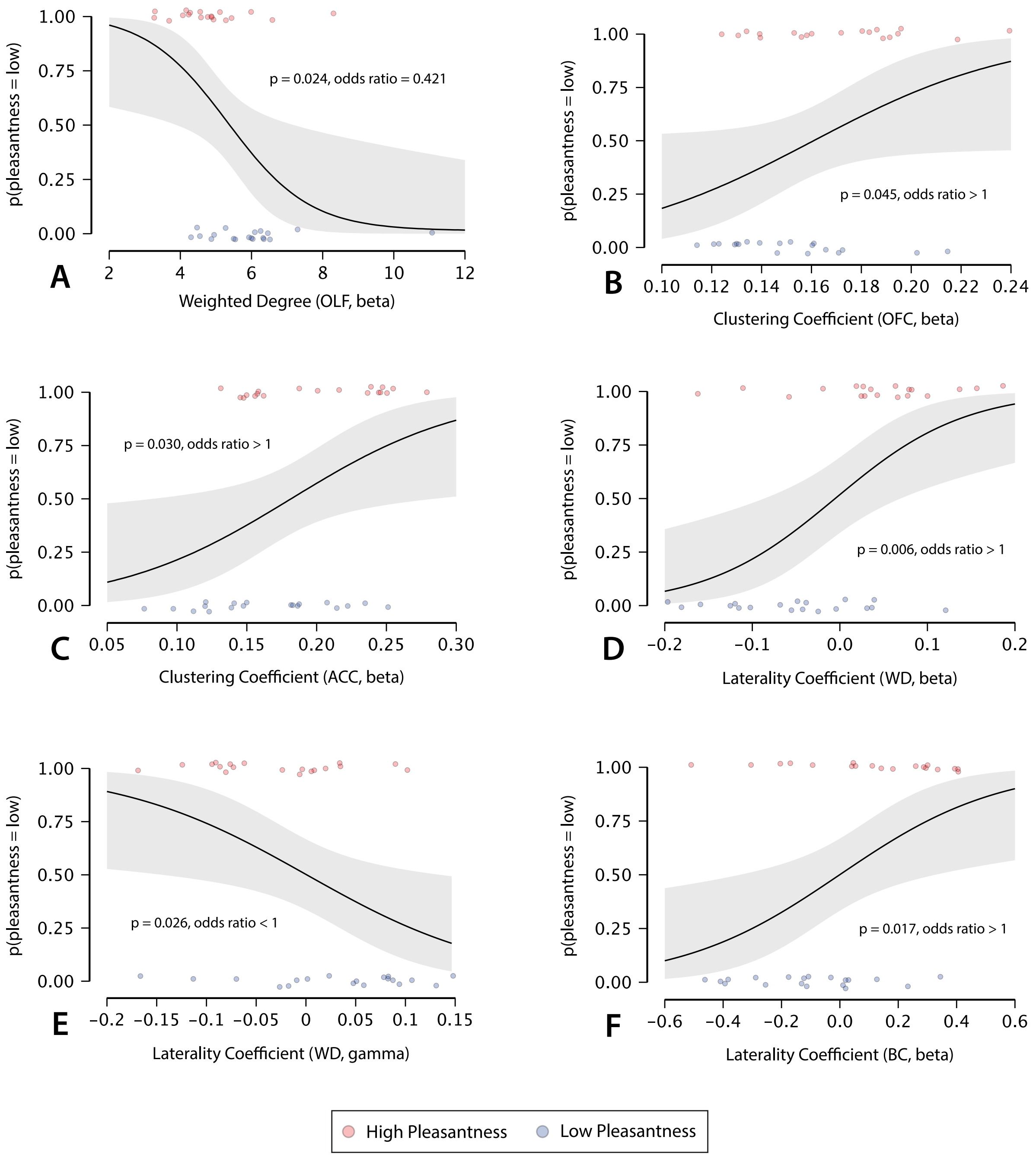

Figure S2. Logistic regression plots showing the probability that a given graph metric or laterality coefficient predicts high/low pleasantness conditions. Analysis was carried out for metrics presented in the main text, figures 4 (panels A to C) and 7 (panels D to F). Each data point represents the value for a single subject (averaged across 10 trials). Odds ratios and p-values are indicated; grey bands show the 95% confidence interval.
